# Plant Perception and Short-Term Responses to Phytophagous Insects and Mites

**DOI:** 10.3390/ijms19051356

**Published:** 2018-05-03

**Authors:** M. Estrella Santamaria, Ana Arnaiz, Pablo Gonzalez-Melendi, Manuel Martinez, Isabel Diaz

**Affiliations:** 1 Centro de Biotecnologia y Genomica de Plantas, Instituto Nacional de Investigacion y Tecnologia Agraria y Alimentaria (INIA), Campus Montegancedo, Universidad Politecnica de Madrid (UPM), Pozuelo de Alarcon, 28223 Madrid, Spain; me.santamaria@upm.es (M.E.S.); a.arnaiz@upm.es (A.A.); pablo.melendi@upm.es (P.G.-M.); m.martinez@upm.es (M.M.); 2 Departamento de Biotecnologia-Biologia Vegetal, Escuela Tecnica Superior de Ingenieria Agronomica, Alimentaria y de Biosistemas, UPM, 28040 Madrid, Spain

**Keywords:** plant defenses, phytophagous arthropods, elicitors, effectors, plant receptors, early signaling, insect, acari

## Abstract

Plant–pest relationships involve complex processes encompassing a network of molecules, signals, and regulators for overcoming defenses they develop against each other. Phytophagous arthropods identify plants mainly as a source of food. In turn, plants develop a variety of strategies to avoid damage and survive. The success of plant defenses depends on rapid and specific recognition of the phytophagous threat. Subsequently, plants trigger a cascade of short-term responses that eventually result in the production of a wide range of compounds with defense properties. This review deals with the main features involved in the interaction between plants and phytophagous insects and acari, focusing on early responses from the plant side. A general landscape of the diverse strategies employed by plants within the first hours after pest perception to block the capability of phytophagous insects to develop mechanisms of resistance is presented, with the potential of providing alternatives for pest control.

## 1. Introduction

Insect and acari pests constitute an important constraint to crop growth and food production. Climate change has a positive effect on herbivore performance, since the increase in temperature shortens their life cycle, speeding up pests’ appearance and increasing the host range [1,2]. Consequently, pest control is one of the major challenges and costs in agriculture today. Efforts in recent years have provided new insights into plant defense responses against phytophagous arthropods, including information about plant molecules with new potential for use as pest control. Plants have developed mechanisms of resistance against pests through coevolution of endogenous defense pathways stimulated by aggressor species. Plant damage varies depending on the nature of the feeder. Plant responses try to overcome each specific injury. Chewing insects, such as caterpillars and beetles, consume large portions of plant tissues, while piercing, sucking insects that feed on the vascular systems of plants, such as aphids, connect their specialized long stylets to the phloem and cause minimal tissue disruption. Thrips combine rasping and sucking methods to feed. Mining-type feeders (larvae of various beetles, flies, and moths) produce snake-like “mines” as they feed between epidermal cell layers within leaf tissues, thus damaged leaves tend to twist and curl [3]. Among phytophagous acari, spider mites of the *Tetranychus* genus (*Tetranychidae*) pierce plant parenchymatic cells using tube-feeding structures to suck their contents, while the short stylet of the tomato russet mite *Aculops lycopersici* (Eriophyidae) allows it to puncture only the epidermal cells [4,5]. Continuous feeding may cause chlorosis, leaf destruction, loss, or defoliation and important alterations in plant growth, development, and yield, and finally lead to severe crop loss. Accordingly, plants respond by adapting their defense against each specific foe to win the battle, depending on the plant species, ecotype, and developmental stage.

**Figure 1:**
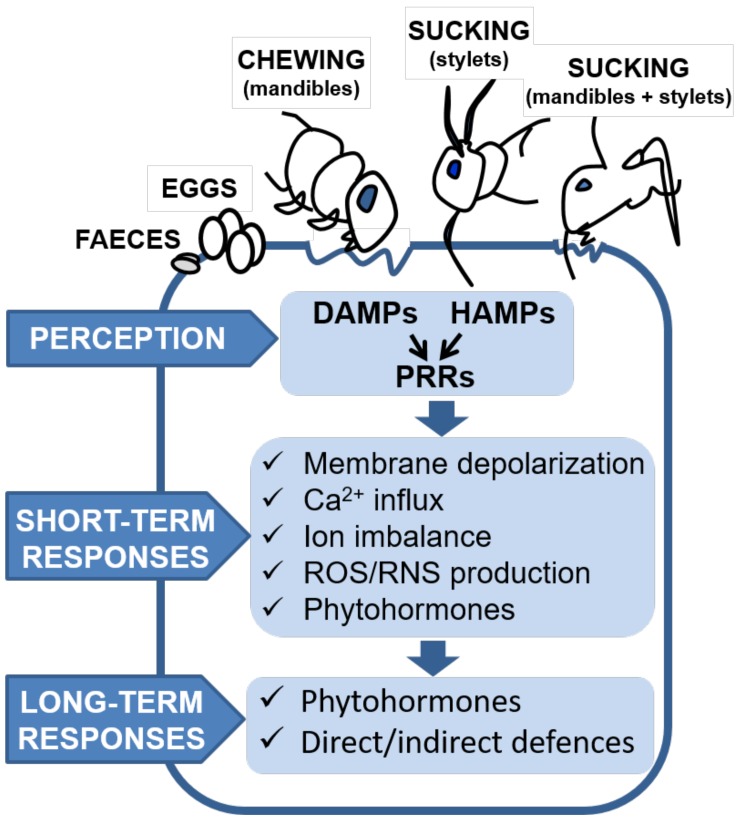
Early and late plant events in plant–phytophagous pest interactions. Insects and acari with different mouth structures and several feeding modes select host plants. Specific plant receptors (pattern recognition receptor, PRRs) recognize elicitors/effectors (damage-associated molecular patterns, DAMPs, and herbivore-associated molecular patterns, HAMPs) derived from either the plant or the phytophagous pest side (insects and acari) and activate downstream short-term and long-term defense responses. ROS, reactive oxygen species; RNS, reactive nitrogen species.

To fight against phytophagous pests, plants have evolved constitutive and inducible defenses, as well as indirect ones using volatiles and nectars to attract natural enemies of insects and acari [6,7]. As part of these defenses, herbivore-challenged plants can also emit volatiles to warn neighboring plants of an imminent threat [8,9]. Subsequently, phytophagous arthropods respond to plant defenses by developing multiple strategies to avoid them [10]. In a second round, plants counter-attack and implement emergency responses [11,12]. The induction of plant defenses is initiated when specific receptors (pattern recognition receptors; PRRs) detect the presence of phytophagous pests by recognizing herbivore-associated molecular patterns (HAMPs), microbe-associated molecular patterns (MAMPs) released with gut-associated endosymbionts by herbivores in their fluids, or damage to plant tissues as a consequence of infestation (damage-associated molecular patterns; DAMPs) [9,13,14,15,16,17]. Plants also detect the presence of volatiles emitted as plant–plant cues and prime defenses against herbivores [8]. Recognition of these molecular patterns activates downstream short-term responses at the membrane level (potential depolarization, Ca^2+^ influxes, and generation of reactive oxygen and/or nitrogen species (ROS and RNS)) [18,19]. Subsequently, a cascade of Ca^2+^-binding protein kinases (CDPKs) as calcium-sensor proteins lead to the synthesis of phytohormones, mainly jasmonic acid (JA), salicylic acid (SA), and ethylene, among others, and the activation of transcription factors that regulate gene expression of a wide range of products. These early events of recognition and rapid response take place within minutes to hours after the initiation of herbivory [18]. In a second step, late-term responses and direct defenses are triggered. These include the release of products with antinutritional, deterrent, repellent, and toxic properties and entrap, inhibit, and interfere with the metabolism, development, and fecundity of phytophagous arthropods [6,7,20,21,22]. Additionally, indirect defenses, mainly volatiles, are produced to attract natural enemies as partners to collaborate in the battle. This repertory of induced pathways contributes to plant survival and mitigates allocation of resources to defense (Figure 1).

The challenges of current biology require integrated multidimensional approaches to delve into the functional complexity of plant defenses against insect attack [23]. Comparative transcriptomic and proteomic profiles of host plants after arthropod infestation or after application of insect oral secretions (OS) have demonstrated that plants discriminate among herbivores and activate specific responses differentially regulated in time and space [24,25,26,27,28]. Metabolomic approaches have corroborated this ability to differentiate pest species and determine the onset of indirect defense responses, mainly volatile emission, to complement the direct ones [29,30]. In addition, some studies have analyzed plant responses to a combination of biotic or biotic–abiotic stresses to mimic interactions in nature. Results highlighted common and divergent features of integrated responses in defense compared to a single stress, providing a deeper understanding of the events involved in these interactions [31,32]. From an applied perspective, characterizing new plant molecules with defense properties and identifying defense-related pathways as potential targets of pest control may provide alternatives for chemical control by using either traditional plant crosses or biotechnological approaches.

This review deals with the molecular events developed by plants within the first hours after pest perception to defend themselves against attacks by insects and mites. The new insight of this review is integration of the different aspects involved in this process, presented step-by-step in sequential order. First, it focuses on the elicitors (that amplify defense response) and the effectors (that amplify attacks). Second, it states the plants’ perceptions of these elicitors and effectors, but also the physical presence of arthropods and the physical damage they produce. Finally, the review describes early cascading reactions as a result of the presence of elicitors/effectors and physical damage, to end with a brief section on pest management based on molecular science as an alternative to conventional chemical control.

## 2. First Signals: Elicitors and Effectors

Plants rely on a battery of sophisticated mechanisms to detect pests in order to trigger appropriate reactions, since their survival depends on a fast response. Time is crucial, and the first step in the early events of defense is perception. Plants may detect highly specific pest-associated cues as well as physical damage and chemical compounds. Interestingly, some effects are perceived and identified by plants as a threat. For example, the high-amplitude vibrations produced by chewing insects while feeding act as an important source of acoustic energy for plants. Appel and Cocroft [33] showed that Arabidopsis plants distinguished *Pieris rapae* (*Lepidotera*, *Pieridae*) chewing vibrations from environmental pulsations. Plants exposed to the caterpillar’s chewing vibrations synthesized greater amounts of chemical defenses, in particular aliphatic glucosinolates, both locally and systemically. Likewise, imprints of *Heliothis virescens* (Lepidoptera, Noctuidae) larvae on upper tobacco (*Nicotiana tabacum*, Solanaceae) surfaces were visualized within seconds through accumulation of chlorophyll and superoxides. The larvae wounded the surfaces of leaves, scratched the leaves with the crotchets on their feet, and stimulated the synthesis of 4-aminobutyrate (GABA) within minutes [34]. It has been suggested that in this early signaling process, GABA might act as a toxin by disrupting the insect’s neuromuscular activity [34].

**Figure 2:**
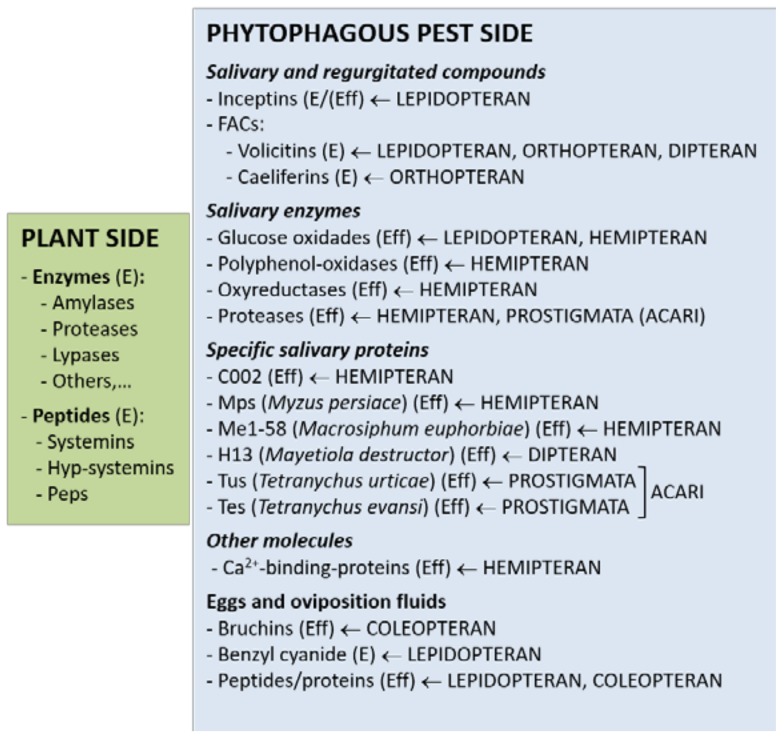
Elicitor (E)/effector (Eff) molecules from plant and phytophagous pest sides trigger, amplify, or suppress specific plant defense responses against pests. Phytophagous insect and acari species and orders are indicated.

When arthropod infestation is initiated, damage of plant tissues can bring inevitable consequences of contact, walking, feeding, oviposition, and feces or frass deposition. Injury produces cell lysis and macromolecule fragmentation, releasing endogenous compounds from disrupted tissues. These compounds, considered as DAMPs, trigger nonspecific plant defense responses providing plant protection, independent of the damaging agent [34]. Additionally, plants induce defenses elicited by a battery of HAMPs directly released by the host plant or the arthropod. HAMPs allow specific recognition of the pest species and induce a precise defense against each particular attacker. However, it is difficult to differentiate and categorize HAMPs and DAMPs, since arthropod infestation stimulates a combination of both simultaneously, providing tools for the plant–pest battle. To make the host–phytophagous pest interface even more complex, some arthropod-derived molecules are secreted by specialized herbivores and overcome the host defense system as a mechanism of counterattack [35,36]. In this context, it is essential to clarify elicitor and effector concepts. Generally, elicitors are molecules able to activate signal transduction pathways for the synthesis of metabolites, which may reduce damage and increase plant resistance against pests. In contrast, effectors are molecules that can selectively either trigger or compromise plant immunity by binding to other molecules and altering the defense machinery. Elicitors come directly from the plant or the arthropod during pest infestation to induce or amplify the defense response either locally or systemically (Figure 2).

### 2.1. Elicitors from the Plant Side

The most abundant elicitor compounds from the host are peptides. Among them, systemins (Sys) are small peptides of 18 amino acids derived from cytosolic precursor proteins of about 200 residues, termed prosystemins. They have been found exclusively within the Solanaceae family in response to wounding, insect feeding, and/or JA treatment [37,38]. The tomato (*Solanun lycopersicum*, Solanaceae) prosystemin, probably the best studied, is accumulated in the cytosol. Once the peptide has been processed, it is transported via an unknown mechanism to the apoplast, where it interacts with a membrane receptor, initiating a cascade of reactions that leads to the generation of early and late plant defenses [39]. Prosystemin-overexpressing tomato plants activated the accumulation of proteinase inhibitors (PIs), among other molecules, which may inhibit the activity of digestive enzymes of the insect gut. In parallel, these tomato plants emitted volatile terpenes as olfactory cues and attracted predators, providing increased resistance to pests [40,41,42]. In their study, McGurl et al. found that prosystemin-silencing plants were deficient in the expression of PI genes and showed greater susceptibility to insect attacks than control tomato plants [43]. Several experiments indicated that in tomato, Sys and JA work in parallel in the same signaling pathway, coordinating the expression of defenses [41,42]. Moreover, grafting assays with tomato JA-defective mutants suggested that Sys regulates the biosynthesis of JA [44]. Another endogenous plant peptide that functions as an elicitor is hydoxyproline-rich systemin (HypSys), 18 to 20 amino acid glycopeptides derived from polyprotein precursors that are post-translationally modified by hydroxylation of proline residues and glycosylated with pentoses [45]. HypSys, identified in the Solanaceae and Convolvulaceae families, also activated genes involved in JA-mediated defense, including PIs and the octadecanoid pathway, offering resistance to pests [45]. HypSys peptides from tobacco, tomato, and black nightshade (*Solanum nigrum*, Solanaceae) induced the synthesis of PIs as tomato Sys did. However, HypSys from *Petunia* sp. (Solanaceae) prompted the expression of a defensive gene, suggesting a defensive role against pathogens in this species. In potato (*Solanum tuberosum*, Solanaceae), HypSys peptide activated PI gene expression and expression of NPR1 and PAD4 cofactors, which are more related to the basal immune system against pathogens [46].

Significant progress in identifying plant elicitors has been achieved with the Pep family (plant elicitor peptides). Peps are protein fragments of 23 to 29 amino acids long derived from the C-terminal end of PROPep precursor proteins. These small molecules bind to Pep receptors (PEPRs) and promote gene expression to control pathogens and pests [34]. Since the identification of AtPep1, the first characterized Pep of *Arabidopsis thaliana* (*Brassicaeae*) that elicited defense against pathogens [47], several Peps have been identified with anti-herbivory properties. This is the case of rapid accumulation of ZmPROPep3 transcripts from maize (*Zea mays*, Poaceae) in response to *Spodoptera exigua* (Lepidoptera, Noctuidae) OS. Exogenous application of ZmPep3 induced the emission of volatiles in maize and the production of deterrent metabolites, both mediated by JA signaling. Consequently, OS treated plants had resistance to *S. exigua* [48]. Likewise, in Arabidopsis transgenic lines expressing the GUS (β-glucuronidase) reporter gene under the control of *At*PROPep3 promoter or under two promoters of AtPep receptors, AtPEPR1 and AtPEPR2, the levels of GUS activity increased in response to chewing and sucking insects. GUS induction was located around the site of herbivore feeding independent of the feeding mode, as well as on specialist vs. generalist feeding behavior [49]. In addition, *pepr1* and *pepr2* double mutant plants displayed reduced resistance against *Spodoptera littoralis* (Lepidoptera, Noctuidae) larvae infestation compared to control. Peps are conserved across plant families, and the cross-species functionality among orthologs as regulators of herbivore-associated volatiles is preserved [44,48].

Another class of plant peptides induced by pests is small molecules termed inceptins. They are cryptic peptides of about 10 to 11 amino acids with a disulfide bound that are contained in the chloroplastic ATP synthase γ-subunit sequence (cATP) of legumes. Inceptins have been found in insect OS once the cATP has been ingested, cleaved in the insect midgut, and processed to produce the active peptide. In the case of cowpea (*Vigna unguiculata*, Fabaceae) derived OS of *Spodoptera frugiperda* (Lepidoptera, Noctuidae), a mixture of inceptin-related peptides with different activities depending on certain conserved amino acids in their sequences has been identified [50]. Cowpea plants respond to the generalist armyworm *S. frugiperda* through detection of these proteolytic digestive cATP fragments. Plants promote the synthesis of defense-related phytohormones such as JA and ethylene and the emission of volatiles to attract natural enemies within the first 30 min to 4 h after ingestion. Conversely, OS of legume-specialized caterpillars did not elicit ethylene production and reduced significantly volatile emissions. Schmelz et al. [12] suggested that specialist insects minimize the activation of defenses by converting an elicitor into an antagonist effector. Moreover, they demonstrated that a single amino acid substitution in the inceptin sequence recovered plant elicitation and defense responses against legume-specialist herbivores.

### 2.2. Elicitors/Effectors from the Pest Side

The group of known HAMPs derived from the pest side includes fatty acid–amino conjugates (FACs), salivary enzymes (β-glucosidases, oxidases, glucose-oxidases, alkaline phosphatases, and proteases), Ca^2+^-binding proteins, and other specific proteins, many of them of unknown function (Figure 2) [51]. The salivary elements and regurgitated compounds from the digestive system may act not only as elicitors to prompt defenses, but also as effectors to suppress the induced defenses. In consequence, a compatible plant–pest interaction is produced unless a second phase of plant defense takes place. FACs are conjugates of unmodified or oxidized derivatives of polyunsaturated fatty acids, mainly oleic, linoleic, or linolenic acid from plant origin, and glutamine or glutamine amino acid from the insect. These plant–pest conjugated forms are synthesized in the insect midgut, developing an important role in N assimilation at the larval stage [52]. The first characterized FAC was the volicitin *N*-(17-hydroxylinolenoyl)-l-glutamine, identified in regurgitants of *S. exigua.* Since then, more volicitins have been found in other lepidopteran species and in dipteran species [52,53]. Alborn et al. [53] showed that the *S. exigua* volicitin in contact with damaged leaves of maize activated the emission of volatile organic compounds and attracted parasitic wasps that prey on larvae. A tritiated form of this volicitin bound with high affinity to plasma membrane fractions isolated from maize, although the putative receptor is still unknown [54]. More recently, Shinya et al. [55] studied FAC composition and content in OS of two chewing insects of rice (*Oryza sativa*, Poaceae) and found high amounts of known FACs, mainly volicitin, in OS of *Mythimna loreyi* (Lepidoptera, Noctuidae), whereas no known FACs were detectable in OS of *Parnara guttata* (Lepidoptera, Hesperidae). However, both lepidopteran OS induced ROS accumulation and secondary metabolite production in rice, considered as early and late responses, respectively. These findings indicate that plants integrate different and specific signals and stimuli from the feeder to modulate the defense response and make it more robust.

In general, the transduction of FAC signal into downstream plant responses is still not completely established. However, work reported by Dinh et al. [56] added a new key player in the FAC-mediated process. They identified a FAC-regulated protein in *Nicotiana attenuate* (Solanaceae) named NaHER1, essential for the signaling pathway to amplify plant defenses in response to OS elicitation. NaHER1 accumulated in the plant after 1 h of *Manduca sexta* (Lepidoptera, Sphingidae) OS treatment, independent of JA. Resulting tobacco *NaHER1* silencing lines were more susceptible to larvae feeding than control plants and had impaired JA signaling and biosynthesis and strongly reduced direct and indirect defenses. In addition, it was demonstrated that the NaHER1 protein acted as a natural suppressor of abscisic acid (ABA) catabolism. The low levels of JA and ABA in tobacco *NaHER1* silencing plants turned in less defense and, as consequence, better larvae performance and a bigger larvae mass when fed on silencing lines compared to control plants. Molecule analogs of FACs are caeliferins, sulfated α-hydroxy fatty acids of 1 to 20 carbons found in OS of several phytophagous insects, most members of the Caelifera suborder within the Orthoptera order. Their physiological function in herbivory is not fully understood, but they probably participate in the digestion process, since their hydrophilic sulfate group may act by bridging lipids and water. These compounds also elicit the release of plant volatiles after feeding or when applied to damaged leaves, which has been shown to result in the attraction of natural enemies [57].

Since the first report demonstrating that glucose oxidase from the saliva of the caterpillar *Helicoverpa zea* (Lepidoptera, Noctuidae) specifically counteracted the production of nicotine [58], many glucose oxidases have been identified in the OS of many caterpillars and aphid species. Their role as suppressors of induced defenses has been clearly demonstrated in several plant hosts [59,60]. Particularly, Diezel et al. [61] showed that a glucose oxidase found in *S. exigua* elicited SA accumulation in *N. attenuata*, which might antagonize JA-mediated host defenses and neutralize them. There are many other examples of molecules identified in OS or in salivary glands with hidden activities to turn off plant defenses [51]. Probably effectors from hemipteran, and particularly from aphids, are the most widely studied. Aphids produce two types of saliva, gelling sheath saliva, which surrounds their stylets and protects them from plant defenses, and watery saliva, which is released into the vascular system of the plant during the phloem-feeding process. Watery saliva contains a variety of compounds, including multiple hydrolytic and detoxifying enzymes and proteins with signal sequences for secretion, either species-specific or adapted to a particular host, with a potential role as effectors. 

The first salivary protein in aphids described as an effector was C002, a specific protein of unknown function with homologs in other organisms not yet identified. Overexpression of C002 from *Myzus persicae* (Hemiptera, Aphididae) in *N. benthamiana* increased aphid reproduction. Conversely, C002 dsRNA either microinjected into *Acerthosiphon pisum* (Hemiptera, Aphididae) or silenced using RNAi technology in plants was lethal for this aphid species and negatively affected *M. persicae* development and fecundity [10,62]. Pitino and Hogenhout [63] reported that this aphid effector may also determine aphid host range. While *M. persicae* C002 overexpressed in Arabidopsis increased the progeny of this species, its ortholog gene from *A. pisum* ectopically expressed in Arabidopsis did not promote *M. persicae* performance. This was in accordance with their host range, since *A. pisum* is restricted to Fabaceae species, whereas *M. persicae* is a polyphagous that easily colonizes Arabidopsis. Advances in genome sequencing and elicitation assays using transient expression assays in *N. benthamiana* have allowed the identification of a wealth of potential aphid effectors with different properties and specificities [64]. As examples, *M. persicae* Mp1 and Mp2 proteins stably expressed in Arabidopsis suppressed plant defenses and enhanced aphid performance. Additionally, Mp55–58 have shown a differential impact on aphids across *N. benthamiana, N. tabacum*, and *A. thaliana* [65]. Likewise, Me10 and Me23 from the potato aphid *Macrosiphum euphorbiae* (Hemiptera, Aphididae) clearly increased aphid fecundity when they were expressed *in planta* [66]. More recently, a novel effector, Me47, found in the saliva secretome of *M. euphorbiae* and identified as a glutathione-S-transferase, enhanced potato aphid fecundity in tomato plants and green peach aphid fecundity in *N. benthamiana*. Interestingly, Me47 expressed in Arabidopsis decreased *M. persicae* fecundity, which corroborates the host range specificity previously reported [67].

Calcium-binding proteins are a group of molecules that interfere with the occlusion of phloem sieve elements mediated by Ca^2+^ in response to aphid feeding [63]. Ca^2+^-binding proteins from the vetch aphid *Megoura viciae* (Hemiptera, Aphididae) neutralized and dispersed specific proteins from bean (*Phaseolus vulgaris,* Fabaceae) involved in occluding sieve elements in response to calcium [68]. Besides, the breakdown of sieve-tube proteins by certain metallo-proteases found in the watery saliva of *M. persicae* and *A. pisum* is another established mechanism of overcoming plant defenses [69]. Polyphenoloxidases, peroxidases, and oxi-reductases have also been found in aphid saliva, presumably to detoxify phenols and ROS [64].

In contrast to the information widely reported on aphid effectors, insect effectors from other orders have been poorly described, although it is known that they also counterattack plant defenses. The study of salivary secreted proteins in dipteran species has almost exclusively focused on the Hessian fly *Mayetiola destructor* (Diptera, Cecidomyiinae), an important gall midge pest of wheat (*Triticum aestivum*, Poaceae). A transcriptomic analysis of salivary glands identified a large portion of putative effectors with no orthologs in other arthropods. Particularly, *M. destructor* avirulent larvae secretes H13, an effector encoded by an *Avr* gene, which is not detected in virulent larvae. H13 RNAi-knockdown saved a small number of H13-a–virulent larvae on H13-resistant wheat plants. Accordingly, the insect uses an effector-based strategy to modulate the development of its host [70,71]. Similarly, phytophagous mites are also able to overcome host–plant defenses. Recent studies combining bioinformatics, in situ hybridization experiments, and feeding bioassays have proven that *Tetranychus urticae* and *T. evansi* (Prostigmata, Tetranichydae) spider mites produce at least two families of salivary proteins with strong suppressor effects on SA-plant defenses that promote mite performance [72,73]. However, more work has to be done to clarify their action and identify their target receptors.

Most phytophagous insects and acari have a short life cycle with different developmental stages, varied in morphology but always starting from an egg. Arthropod eggs represent a potential threat for plants. Therefore, priming of defenses anticipated for larval hatching and feeding will provide advantages for plant development and survival. While eggs of some arthropod species, such as Tetranychidae mites, do not produce physical injury to plants, eggs from other phytophagous arthropods induce lesions on plant tissues, generate neoplasms, or even form galls. Egg depositions, with or without tissue damage, are perceived by plants and trigger specific responses. Moreover, the perception of eggs primes plant defenses against herbivory [15]. Larvae feeding on egg-deposited plant tissues weigh less, and even suffer higher mortality, than larvae feeding on egg-free plants [15]. It is known that the presence of compounds in secretions associated with the eggs (elicitors) prompts plant responses, which result in the emission of leaf volatiles that attract egg parasitoids [15]. At present, only some eggs and oviposition fluid-associated HAMPs have been characterized. Among them, long-chain fatty acids esterified with 3-hydroxypropanoic acids, termed bruchins, have been identified in eggs of beetles, particularly in *Bruchus pisorum* and *Callosobrochus maculans* belonging to the Bruchinae subfamily within the Coleoptera order [74]. These molecules elicit the formation of tumor-like structures in legume pods, making them difficult for larvae to penetrate. Additionally, oviposition fluids from *B. pisorum* on Brussels sprout leaves modify leaf chemicals, attracting egg parasitoid wasps [75]. In contrast, the presence of benzyl cyanide, a male-derived antiaphrodisiac, identified in the oviposition fluids of *Pieris brassicae* (Lepidoptera, Pieridae), induced direct and indirect defenses when applied to *Brassica oleracea* (Brassicaceae) and *A. thaliana* leaves [76,77]. In oviduct secretions of lepidopteran and coleopteran species, elicitor molecules of proteinaceous nature have also been found that trigger the emission of a repertoire of volatiles to attract egg enemies [14].

Alternatively, phytophagous pests may use egg-mediated effects for their own benefit, since egg effectors may have a role in detoxification, digestion, and suppression of host defenses [15]. Treating plant with extracts of crushed insect eggs has been shown to result in suppression of defense against generalist herbivorous larvae. The suppression of defense was linked with accumulation of SA and downregulation of JA-mediated defense. This finding indicates the presence of an effector inside insect eggs [78].

In recent years, studies have demonstrated that insect frass, considered as HAMPs, and herbivore-associated endosymbionts, considered as MAMPs, also play a role in modulating plant defenses [17,79]. Particularly, insect gut-associated endosymbionts, described in chewing coleopteran and lepidopteran and piercing-sucking hemipteran species, induce physiological changes and may up- or downregulate complex plant defense signaling pathways. There are examples of these symbiotic bacteria being identified in insect OS and developing a role in the activation of SA pathway by suppressing JA-mediated defenses. Alternatively, some endosymbionts are required for induction of antiherbivore molecules, but generally, herbivore bacterial communities are influenced by the host plant [17,80,81]. Likewise, the presence/absence of the symbiotic bacteria *Spiroplasma, Cardinium*, and *Wolbachia* in different *T. urticae* strains has been demonstrated to have an important effect on mites’ host plant by altering distinct plant defense parameters, and affect mite performance [82]. Similarly, the functions of nine facultative symbionts in aphids have been studied, one by one, and results demonstrated that they may affect aphid fitness and evolution and the interactions of aphids with their hosts [83].

In conclusion, the type and combination of elicitors/effectors and physical damage modulate plant defenses to control pests. Although the number of identified elicitors/effectors from both the plant and arthropod sides seems high, new members involved in specific plant–pest relationships will be discovered in the future. This idea is based on the fact that more than half of the known million insect species are phytophagous, and Tetranychidae (spider mites), Tenuipalpidae (false spider mites), and some Eriophyoidae mites are plant feeders [5]. However, few arthropod species have been studied. Besides, knowledge on the role of herbivore-associated endosymbionts in determining plant defense responses is still limited. In this scenario, the most challenging aspect is to identify new signals and understand how these signals are recognized by plants to trigger a cascade of defenses and counter-defense effects.

## 3. Plant Perception

Plants have developed good perception systems for phytophagous threats that allow rapid distinction between physical injury and pest compounds. Plant-specific receptors (PRRs) detect and recognize either the presence of elicitors/effectors (HAMPs) or the damage (DAMPs) produced by phytophagous insects and acari as the first step of triggering defense. Generally, PRRs are encoded by multigene families and are located at the plasma membrane, with additional ectodomain and cytosolic domains required to start a specific recognition, followed by a transduction of defense signaling pathways. PRRs can be either receptor-like kinases (RLKs) or receptor-like proteins (RLPs). RLKs are composed of an ectodomain potentially involved in ligand binding, a transmembrane region, and an intracellular kinase domain. RLPs have a similar structural organization, but lack the intracellular kinase domain [84].

Many reports about the characterization of PRRs of PAMPs have been published, but only very few HAMP–PRR interactions have been characterized. Plant perception of phytophagous arthropods has been particularly investigated in the case of lepidopteran and aphids. In 2004, Truitt et al. [54] provided the first experimental evidence of selective reversible binding between enriched plasma membrane fractions of *Z. mays* and a volicitin from *S. exigua.* This elicitor-unknown receptor binding activated the emission of volatiles by attracting female parasitic wasps that prey on herbivore larvae. Gilardoni et al. [85] showed that the FAC 18:3Glu identified in the larval OS of *M. sexta* bound to LecRK1, a lectin-receptor kinase of *N. attenuata. LecRK1* mRNA levels increased at 1 h of *M. sexta* 18:3Glu treatment, and it was also elicited by OS from other lepidopteran species. Both virus-induced *LecRK1* silencing and RNAi *LecRK1* knockdown lines in *N. attenuata* demonstrated that this receptor was essential to induce the synthesis of defense compounds such as nicotine, diterpene-glucosides, and trypsin protease inhibitor. Moreover, *LecRK1* participated in the suppression of the insect-mediated inhibition of JA-induced defense responses, since silenced *LecRK1* lines highly increased the accumulation of SA and reduced JA and JA–Ile content in response to *M. sexta* OS treatment.

Other examples of well-characterized plant receptors of HAMPs are the tomato receptor-like kinase SISERK1, the *A. thaliana* l-type lectin-receptor kinase LecPRK-1.8, and LRR receptor-like kinase BAK1 (brassinosteroid insensitive-associated kinase) [86,87,88,89]. SISERK1 is required for the full action of the nucleotide binding leucine-rich repeat resistant protein Mi-1 to confer resistance against aphids and nematodes [86]. *SISERK1* was identified in a virus-induced gene silencing screen in *N. benthamiana* based on the suppression of plant responses triggered by a constitutively active form of Mi-1. Recently, co-immunoprecipitation (CoIP) assays combined with confocal microscopy have shown that SISERK1 and Mir-1.2 were present in the protein complex at the plasma membrane and the conformation of the complex was altered by potato aphid-derived saliva [87]. Tomato plants overexpressing *SISERK1* displayed enhanced resistance to aphids. LecPRK-1.8 is upregulated in response to egg-derived elicitors of the lepidopteran *P. brassicae.* LecRK-1.8 T-DNA plant insertion lines reduced the induction of the defense PR-1 expression under treatment of the nonpolar fraction of *P. brassicae* eggs compared with Col–WT lines. These results indicate that the interaction between LecRK-1.8 with specific egg-derived elicitor(s) was crucial for the early detection and subsequent induction of plant defenses [88]. BAK1 is required to activate the defensive response against the aphid *M. persicae*, including aphid elicitor–mediated induction of reactive oxygen species and callose deposition. Fractions derived from extracts of the aphid were able to trigger induced resistance in wild-type plants but not in Arabidopsis *bak1* mutant plants. Arabidopsis *bak1* plants are also compromised in immunity by the aphid *A. pisum*, for which Arabidopsis is not normally a host [89].

In addition, some receptors are involved in the perception of plant molecules released upon herbivory [90]. Plant elicitor peptides (Peps) are potent inducers of the immune response against pathogens and herbivores that bind LRR receptors termed PEPRs [91,92]. In some plant species, both Peps and their cognate PEPRs are induced by chewing lepidopteran, sucking aphids, and thrips, suggesting a conserved mechanism of the Pep–PEPR system for herbivore defense [45]. *S. littoralis* feeding bioassays on *pepr1* and *pepr2* mutant Arabidopsis plants impaired AtPep signaling, leading to slower production of JA and increased larval weight [49,50]. However, Pep signaling in the context of phytophagous pests requires further investigation. As previously mentioned, Sys triggers systemic defense responses in plants after being wounded or attacked by phytophagous pests [37]. In tomato, perception of Sys depends on SYR1, an LRR-RK that binds Sys with high affinity and specificity [93]. An introgression line of *S. lycopersicum* with a specific part of its genome replaced by the homologous part of the wild tomato species *Solanum pennellii* (Solanaceae) lacked responsiveness to Sys. Larvae of the generalist herbivore *S. littoralis* feeding on plants from this line performed better and gained significantly more weight than the ones that fed on tomato wild-type, which corroborates that Sys perception contributes to the resistance of tomato plants against herbivory [93]. In addition, ethylene receptors are membrane-bound proteins with histidine kinase activity and associated with a mitogen-activated protein (MAP) kinase downstream signaling pathway [94]. As ethylene is a molecule largely involved in herbivore signaling [95], these receptors may be considered as part of the system of perception of phytophagous pests.

In summary, the dynamic association of plant PRRs with specific elicitors/effectors has been shown to effectively mediate resistance against phytophagous arthropods. Despite the high number and different types of characterized PRRs involved in plant–pathogen detection and substrates that link PRR activation to the induction of early signaling, these are just some examples demonstrating this interaction of plant PRRs in plant–pest interactions. Understanding PRR organization and activation and the subsequent connection to downstream signaling networks is the main challenge. Mostly, PRR kinases and phosphorylation events seem to mediate signaling initiation related to PRR-elicitor/effector recognition. Deciphering specific signaling domains in the receptor and determining the fate of the activated PRRs not only highlights the first steps in detecting the presence of specific phytophagous species, but elucidates the branching of the signaling from the PRR complex.

## 4. Early Plant Signaling Events

**Figure 3:**
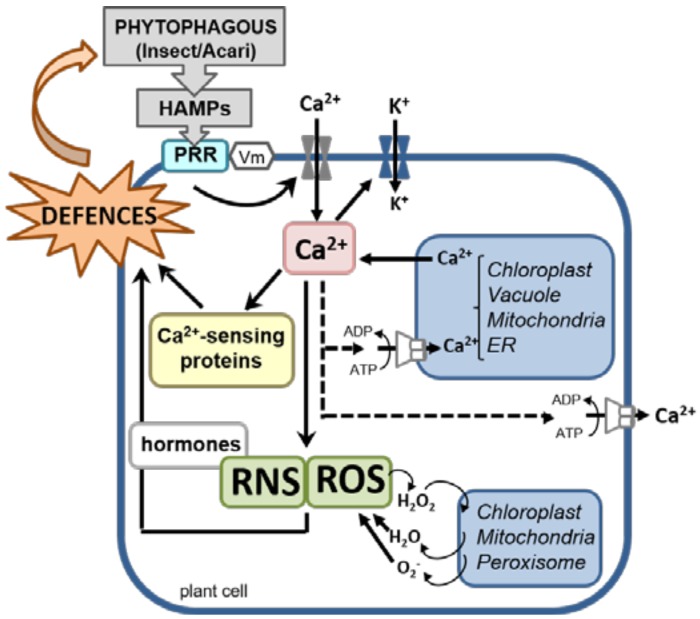
Early plant events in plant–phytophagous pest interactions. Specific plant receptors (PRRs) recognize elicitors/effectors (HAMPs) derived from either the plant or the phytophagous pest side (insects and acari) that induce alterations in the membrane potential (Vm) and cytosolic Ca^2+^ influxes from the apoplast and organelles. Subsequently, Ca^2+^-sensing proteins are activated and reactive oxygen species (ROS) and reactive nitrogen species (RNS) burst is triggered. Then cytosolic Ca^2+^ concentration is restored via Ca^2+^-ATPases, and finally, in a second step, specific hormone-mediated defenses against pests are produced.

The perception of HAMPs rapidly activates a cascade of short-term responses, starting at the plant cell plasma membrane, that eventually result in the development of specific defenses (Figure 3). Phytophagous pests and pathogens induce depolarization in the membrane potential (Vm), followed by fast electrical signals that act as the first alert message for the whole plant. Vm depolarization is correlated to cytosolic Ca^2+^ influxes from the apoplast and some organelles, ion channel activities (mainly opening of K^+^ channels), and ROS and RNS production. Although chemical signals travel much slower than electrical signals within the plant, all these actions take place seconds or minutes after pest detection [17,90]. Calcium ions act as second messengers and activate Ca^2+^-sensing proteins, including calmodulins, calmodulin-like and calcineurin B-like proteins, and CDPKs. This is followed by MAP kinase (MAPK) participation, phytohormone synthesis, and activation of transcription factors that regulate the gene expression of defense compounds. Arimura et al. [96] demonstrated the inhibitory effect of the extracellular Ca^2+^ chelator BAPTA (1,2*bis*-2-aminophenoxy-ethane-*N*,*N*,*N*′,*N*′-tetraacetic acid) on the synthesis of defense compounds in *T. urticae*–infested lima beans (*Phaseolus lunatus*, Fabaceae) and in leaves exposed to plants emitting *T. urticae*–induced volatiles. Qiu et al. [97] reported that Ca^2+^/calmodulin binding to the transcription factor AtSR1 was indispensable for *Bradysia impatiens* (Diptera, Sciaridae)-induced response, since it suppressed SA accumulation. Moreover, *atsr1* mutant plants were more susceptible to *B. impatiens* flies than wild-type plants. Likewise, different CDPKs from soybean exhibited specific expression patterns, up- or downregulated, after the chewing of *S. exigua* or the piercing-sucking of *Aphis glycines* (Hemiptera, Aphididae), independently on hormonal signaling [98]. Silencing *N. attenuata* CDPK4 and CDPK5 strongly upregulated *M. sexta*–induced JA accumulation [99]. Some reports have also shown MAPKs as a group of enzymes involved in the early events induced in the plant–phytophagous arthropod interaction. Tomato LeMPK1, LeMPK2, and LeMPK3 participated in the systemin-mediated defense response against *M. sexta.* The co-silencing of LeMPK1 and LeMPK2 compromised prosystemin resistance to this lepidopteran [100].

Once the Ca^2+^-signaling pathway after phytophagous pest attack is ongoing, the initial cytosolic Ca^2+^ concentration is restored, avoiding harmful effects. For this, Ca^2+^-ATPases mediate calcium efflux to apoplast, vacuole, mitochondria, and endoplasmic reticulum [17,101]. Additionally, the perception of phytophagous and membrane depolarization triggers ROS and RNS in many plant species, but the understanding of oxidative and particularly nitrosative signaling is still very limited. ROS/RNS induce chemical oxidations of some molecules, either activating defense actions against stresses or, alternatively, acting as toxic molecules with strong oxidant power. The final consequence depends on the regulation of the redox status balance in the cell, since moderate ROS/RNS concentrations may differentially sense defense signaling. Conversely, an excess of oxidative stress results in programmed cell death. This dichotomy depends on ROS/RNS accumulation, which is modulated by the complex equilibrium between ROS generating/scavenger systems [102,103,104,105].

ROS levels, especially H_2_O_2_, increase during insect and acari feeding or egg deposition [17,103,105,106,107]. Production of H_2_O_2_ is determined by the activity of oxidases, including salivary glucose oxidases from the phytophagous pest, and several plant oxidases, mainly NADPH oxidases known as respiratory burst oxidase homologs (RBOHs), localized at the plasma membrane of the plant [17,108,109]. RBOHs were rapidly induced in *N. attenuata* and *A. thaliana* plants by OS containing FACs from different lepidopteran species. In addition, *NaRBOHD*-silenced lines were more susceptible to *S. littoralis* feeding [108,109]. Chemical inhibition of the activity of ROS-generating RBOHs in wheat plants infested with the aphid *Diuraphis noxia* (Hemiptera, Aphididae) not only inhibited the accumulation of H_2_O_2_ in leaves, but also prevented the induction of defensive compounds [110]. Likewise, *S. littoralis* induced the accumulation of NO levels in lima beans, as the brown planthopper *Nilaparvata lugens* (Hemiptera, Delphacidae) did in rice leaves after 1 h of infestation. The time course of NO production was parallel to the defense volatile emission observed in lima beans. Exogenous application of NO reduced the injury produced by the plant hopper feeding [111,112]. Interestingly, Wunsche et al. [113] showed that silencing GSNOR, an *S*-nitrosoglutathione reductase, in *N. atteanuata* plants, displayed higher susceptibility to *M. sexta* feeding by impairing JA and ethylene accumulation. In consequence, JA-inducible responses were compromised and defense-related secondary metabolites used for insect control were diminished. Despite these observations, the physiological significance of ROS and RNS in plant defense against pests remains to be established.

ROS/RNS signaling is closely related to hormone signaling, as indicated above. Generally, JA regulates defenses against chewing insects and SA-regulated responses are induced by sucking-feeding insects [114,115], while defense of sucking mites is modulated by fine-tuned regulation between JA and SA [23,107]. Other findings have highlighted the function of other phytohormones such as ethylene, abscisic acid (ABA), auxins, cytokinins, and brasinosteroids [116]. Although phytohormones are mostly elicited as a part of rapid response, they are transduced into signal activation and transcriptional regulation of defense genes. These last events, essential for the generation of direct and indirect defenses, can be considered as a subset of late-term response and need hours or even days after pest detection to complete the defense processes (Figure 1 and Figure 3).

Apart from the inducible defenses that directly target the arthropod physiology, the early plant responses to herbivory are significantly affected by prior experiences. Those plants that experience phytophagous pests or diseases or perceive signals of a forthcoming attack become primed. These plants are now able to respond more quickly than unchallenged plants. In this review, examples of mixtures of volatiles released by plants to attract natural enemies of the phytophagous arthropods have been mentioned as a priming system of protection. Additionally, exposure to a set of stimuli may prepare plants for an upcoming stress event. The end of this priming behavior by airborne signals, mainly green leafy volatiles, is the activation of defense responses in nearby plants [117,118]. Since the first report on the signaling of plant–pest interactions published in 1983 [119], numerous publications have evidenced this defense mechanism. Pearse et al. [120] reported the interplay of volatile signaling in willows as a revisiting of the original “talking trees” by Baldwin and Schultz [119]. Willows (*Salix* spp., Salicaceae) exposed to volatiles from a conspecific neighbor damaged by various folivores experienced less damage than willows lacking a damaged neighbor. The perception of volatile signals from genetically identical clones was more effective at reducing foliar damage to a neighbor than signals from a genetically different individual. Besides, priming can enhance resistance across generations. Ali et al. [121] provide an epigenetic basis of the memory of volatile-mediated habituation for priming antiherbivore responses. Rasman et al. [122] demonstrated the transgenerational priming of jasmonic acid–dependent responses and the role of small interfering RNAs in this process. However, future studies will provide new insights to understand how plants benefit from these spatial and temporal communications.

To summarize, early events occur at the beginning of the cascade of signal transduction after plants recognize herbivores, eventually leading to direct and indirect defenses. These first actions ensure quantitative, timely, and coordinated plant defense responses. Identifying and characterizing new compounds involved in signaling pathways and, more importantly, integrating signal perception and transduction are essential for plant survival. Further research to increase our knowledge might provide new breakthroughs that could be used to develop tools to protect plants for pest control.

## 5. Integrated Pest Management Practices

Under natural conditions, important threats to crop production are mediated by single pests or mixtures of pests, either simultaneously or sequentially. More severe losses are produced when arthropod pests share infection with pathogens or with abiotic stresses. Traditionally, preventive treatments using chemical insecticides and acaricides with specific mechanisms of action have been combined with biocontrol systems to protect plants from insect and mite feeders [123]. Considering the potential harmful impact of synthetic pesticides on the environment and human health, alternative strategies are needed for pest control [124]. Derived from basic research, some elicitors isolated from plants and arthropods, or contained within plant extracts or purified as recombinant molecules, are the most promising molecules to activate plant defense responses to pathogens and pests [125,126,127]. Once more, elicitors such as chitosan, flagellin, and several phytohormones are examples from the large list of compounds included in commercial formulations already proven to control pathogens [125,126]. However, exogenous elicitors in outdoor trials often confer variable and incomplete protection, different than the efficacy observed in laboratory experiments or greenhouses. Transferring this technology to the field requires more investigations. At present, the integration of elicitors in pest management practices is still limited. In contrast, the chemical communication system developed among plants, pests, and predators is being used in biocontrol [128,129]. The future of volatile application on crops will be based on generating improved synthetic products based on identifying natural ones, producing natural metabolites via transgenic plants, or using bioreactors to produce huge amounts of natural compounds and recombinant molecules. In conclusion, generating multidisciplinary knowledge in the plant–pest interphase is essential to integrating a combination of strategies for pest management practices.

## 6. Concluding Remarks

Recent works have enhanced the understanding of mechanisms by which plants are able to recognize phytophagous pests and subsequently activate short- and long-term defense responses. Herbivore infestation simultaneously combines the production of HAMPs, MAMPs, and DAMPs, and plants discriminate among them and induce precise defenses against each particular attacker. In addition, these plant–pest interactions imply higher levels of complexity, since some herbivores secrete arthropod-derived molecules, which have been shown to overcome the host defense system as a counter-attack mechanism. Finally, plant defenses and insect/acari counter-defenses involve adaptations and metabolic costs, so most plant–pest interactions reach a standoff, where both host and pest survive although their development is suboptimal [18]. However, between the perception and final responses from both sides, plant and pest, many questions remain unanswered. What other molecules contained in eggs, feces, and pest fluids might elicit plant defenses? What other PRRs differentially recognize pest elicitors and effectors? How is the signal transduction pathway initiated and what other molecules are involved? How is redox status balance controlled and hormone crosstalk established to regulate downstream defenses? What are the molecular bases of pest-induced plant responses? Hopefully, in the coming years further analyses and studies will integrate pest elicitor/effector–PRR interactions, signaling compounds, and pathways to understand these biological relations and apply the knowledge to enhance the performance of agriculturally important crops.

## Abbreviations

cATP	chloroplastic ATP synthase γ-subunit sequence
CDPK	Ca^2+^-binding protein kinase
DAMP	damage-associated molecular pattern
FAC	fatty acid–amino conjugate
GABA	4-aminobutyrate
HAMP	herbivore-associated molecular pattern
HypSys	hydoxyproline-rich systemin
JA	jasmonic acid
MAMP	microbe-associated molecular pattern
MAP kinase	mitogen-activated protein kinase
OS	oral secretion
Pep	plant-elicitor peptide
PEPR	Pep receptor
PRR	pattern recognition receptor
ROS	reactive oxygen species
RNS	reactive nitrogen species
SA	salicylic acid
Sys	systemin
